# Sustained response to minimal-dose tagraxofusp in a patient with BPDCN and advanced chronic kidney disease

**DOI:** 10.1007/s00277-026-06794-8

**Published:** 2026-01-15

**Authors:** Christina Brummer, Katja Evert, Felix Keil, Jakob Schmidt, Matthias Grube, Wolfgang Herr, Markus Radsak, Stephanie Mayer

**Affiliations:** 1 Department of Hematology and Oncology, University Hospital of Regensburg, Regensburg, Germany; 2 Department of Pathology, University Hospital of Regensburg, Regensburg, Germany; 3 Department of Hematology and Oncology, Hospital of Deggendorf, Deggendorf, Germany

**Keywords:** Blastic plasmocytoid dendritic cell neoplasm, Tagraxofusp, CD123, IL-3, Diphteria toxin, MDS

## Abstract

Blastic plasmacytoid dendritic cell neoplasm (BPDCN) is an extremely rare hematologic malignancy with an aggressive course and poor prognosis. Treatment remains challenging particularly in patients who are ineligible for stem cell transplantation due to resistance to conventional chemotherapy. The introduction of tagraxofusp, a CD123-directed cytotoxin, has significantly expanded therapeutic options and improved outcomes for patients with BPDCN. However, its use can be accompanied by notable adverse events, especially capillary leak syndrome, underscoring the need for careful patient selection and monitoring. Up to date, no data is available regarding the safety of tagraxofusp in patients with chronic kidney failure and cardiovascular co-morbidities. We present the case of a 79-year-old male who developed a solitary, rapidly progressing skin lesion on his lower back. The lesion represented the first manifestation of BPDCN with bone marrow infiltration and concomitant myelodysplastic syndrome (MDS). Molecular analysis identified mutations in CBL, TET2, ZRSR2 and KRAS. Non-eligible for stem cell transplantation, the patient was admitted to treatment with tagraxofusp in a dose-reduced protocol due to concomitant chronic kidney disease (CKD). After three doses of the first cycle, treatment needed to be stopped due to acute-on-chronic renal failure. After treatment disruption, kidney failure was completely restituted to pre-treatment levels. Notably, skin and bone marrow biopsies demonstrated a dermatologic complete response and partial remission of bone marrow infiltration. A watch and wait concept was followed, and prolonged therapy response was obtained for 8 months before relapse. To our knowledge, this is the first reported case demonstrating the use of tagraxofusp in a patient with BPDCN and advanced chronic kidney disease, showing that even a minimum of tolerated treatment dose can induce a sustained response. Despite the risk of adverse events, tagraxofusp should be considered a viable treatment option for elderly patients with poor performance status and significant comorbidities who are ineligible for intensive chemotherapy or stem cell transplantation, as even limited exposure may achieve meaningful clinical responses.

## Introduction

BPDCN is an extremely rare, aggressive hematologic malignancy derived from precursors of plasmacytoid dendritic cells and is associated with poor prognosis due to its rapid progression and limited response to conventional chemotherapies [1]. It typically presents with skin lesions— often violaceous nodules or plaques— and may involve the bone marrow, lymph nodes, and peripheral blood [2]. The disease predominantly affects older adults, with a median age at diagnosis of around 70 years and is associated with poor prognosis due to its rapid progression and limited response to conventional chemotherapies [1]. Immunophenotypically, BPDCN is characterized by co-expression of CD4, CD56, and CD123 while lacking lineage-specific markers for T-, B-, or myeloid cells [3, 4]. For years treatment options were limited, often relying on intensive chemotherapy regimens borrowed from acute leukemia protocols [5].

The approval of tagraxofusp—a CD123-directed cytotoxin—has significantly expanded the therapeutic landscape, offering a targeted approach, particularly beneficial for older patients who are not candidates for stem cell transplantation or aggressive chemotherapy [6, 7]. However, tagraxofusp is associated with a significant risk of capillary leak syndrome (CLS) – a potentially life-threatening adverse event characterized by increased vascular permeability, leading to hypotension, edema, hypoalbuminemia, and hemoconcentration [8]. The pathophysiology involves endothelial damage and fluid extravasation into interstitial compartments [9].

Elderly patients, especially those with cardiovascular disease or chronic kidney disease (CKD), are particularly vulnerable to complications arising from CLS, since the rapid fluid shifts and volume overload can exacerbate chronic heart or kidney failure [10]. Given these risks, the administration of tagraxofusp in such patients necessitates detailed risk-cost evaluation, individualized dosing strategies, close laboratory and clinical monitoring and a proactive approach to early signs of CLS [9]. Up to now, the efficiency and management of tagraxofusp therapy in high-risk patients has been not well-established in the literature [8, 10]. Here, we report the case of an elderly patient with BPDCN and concomitant MDS, presenting with CKD and cardiovascular co-morbidity, in whom initiation of tagraxofusp therapy—administered as a reduced, half-cycle regimen, which represented the maximum tolerated dose—resulted in a prolonged complete remission.

## Case description

**Fig. 1:**
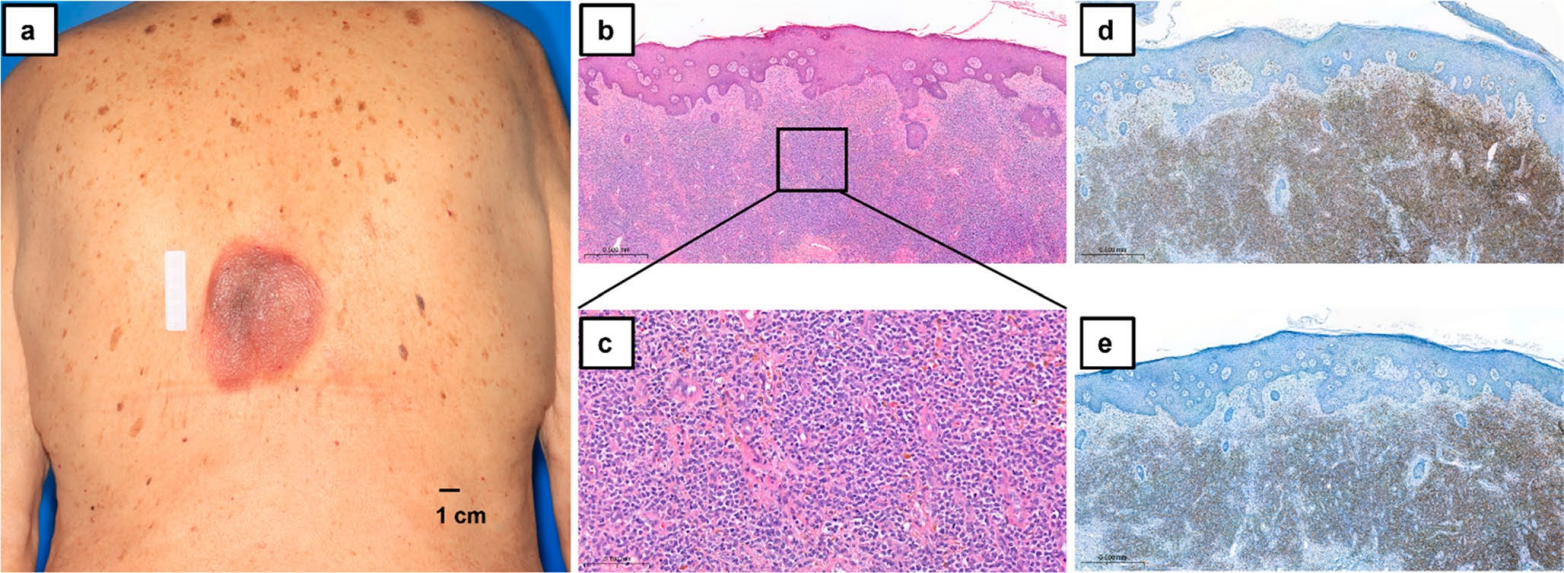
Macroscopic and microscopic imaging of the skin lesion at initial patient presentation. (**a**) Clinical examination revealed confluent plaques, measuring 10 × 8 cm, on the lower back. (**b**, **c**) Hematoxylin and eosin (HE) staining (Magnification b: 20x and c: 200x) of the skin biopsy showing diffuse basophilic blast infiltration. (**d**, **e**) The blasts show consistent staining for (**d**) CD123 and (**e**) CD56 (Magnification: 20x)

A 79-year-old male was admitted to our hospital in July 2024 due to a rapidly progressing skin lesion on his lower back. The patient reported the onset of spontaneous, non-pruritic erythema on his lower back two weeks before, which progressively evolved into ulcerative plaques. His medical history was notable for preexisting CKD, arterial hypertension, and atrial fibrillation but he denied any history of trauma, diabetes, or underlying rheumatic or infectious conditions. Upon clinical examination, confluent plaques, measuring 10 × 8 cm, were observed on the lower back (Fig. 1a). Laboratory tests revealed bicytopenia, characterized by hyperchromic macrocytic anemia and thrombocytopenia. Kidney function was impaired, as evidenced by elevated creatinine (1.62 mg/dL) and a reduced glomerular filtration rate (GFR 40 mL/min), consistent with preexisting CKD (G3A2). Other routine tests, including transaminases, electrolytes, lactate dehydrogenase, and inflammatory markers such as C-reactive protein were within normal range.

**Fig. 2:**
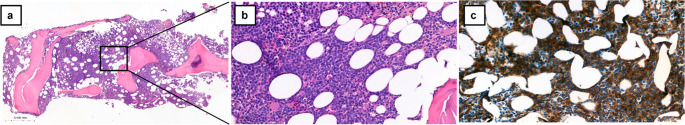
Microscopic imaging of the bone marrow biopsy at initial patient presentation. (**a**, **b**) HE staining (Magnification a: 20x and b: 200x) of the hypocellular bone marrow biopsy showing diffuse basophilic blast infiltration with (**c**) consistent staining for CD123 (Magnification: 200x)

To further investigate, a punch biopsy was performed, revealing dense, diffuse dermal infiltration with CD56 and CD123 positive blasts, characteristic of BPDCN (Fig. 1b-e). PET-CT, cMRI and a lumbar puncture revealed no other evidence of disease, especially no lymph node or organ involvement. Regarding bicytopenia, a bone marrow biopsy was perfomed, which showed 5% infiltration by BPDCN cells (Fig. 2). Additionally, a significantly dysplastic megakaryopoiesis and erythropoiesis was noted in the hypocellular marrow, suggesting the presence of concurrent myelodysplastic syndrome (MDS). Cytogenetic analysis demonstrated an abnormal karyotype (45,X,-Y), and molecular testing identified mutations in CBL, KRAS, TET2, and ZRSR2.

Due to the patient’s advanced age and significant comorbidities, he was deemed ineligible for allogeneic stem cell transplantation. After evaluation in a multidisciplinary tumor board, treatment with the CD123-targeted cytotoxin tagraxofusp was initiated. Given the lack of available data regarding the safety and dosage of tagraxofusp in patients with impaired renal function and limited cardiac reserve, the treatment regimen was discussed with the pharmaceutical company prior to initiation and closely monitored in a controlled inpatient setting. To mitigate the risk of treatment-related complications—particularly capillary leak syndrome—the standard tagraxofusp dosing regimen (12 µg/kg daily on days 1–5) was modified to an intermittent schedule with administration every other day. This adjustment was implemented to allow for improved physiological recovery between doses and was accompanied by intensive monitoring of renal function, fluid balance, serum albumin levels, and body weight. The every-other-day schedule was chosen based on evidence from the pivotal approval trial demonstrating preserved therapeutic efficacy with adjusted dosing intervals, whereas dose reduction lacked supporting data for effectiveness [11].

However, after three of the five planned tagraxofusp infusions of the first cycle (d1, d3, d5), the patient developed acute-on-chronic renal failure, evidenced by anuria and progressive increase in serum creatinine levels from day 7. As a result, tagraxofusp treatment was immediately suspended. Given the possibility of CLS as an underlying condition for acute worsening of kidney function, adjunctive therapy with prednisolone at a dose of 1 mg/kg body weight was initiated. On day 10 the kidney function parameters peaked and subsequently improved toward baseline levels, so that the patient could be discharged in stable condition a few days later. Follow-up visits in the oncological outpatient clinic four weeks later showed that renal function had returned to pre-treatment levels, and macroscopically the skin lesion had nearly resolved. Consolidating low-dose radiation therapy (2 × 4.0 Gy) was initiated for the residual skin lesion. A subsequent punch biopsy confirmed complete histopathological remission of skin infiltration, with no residual evidence of BPDCN, while bone marrow assessment revealed only minimal residual infiltration.

**Fig. 3:**
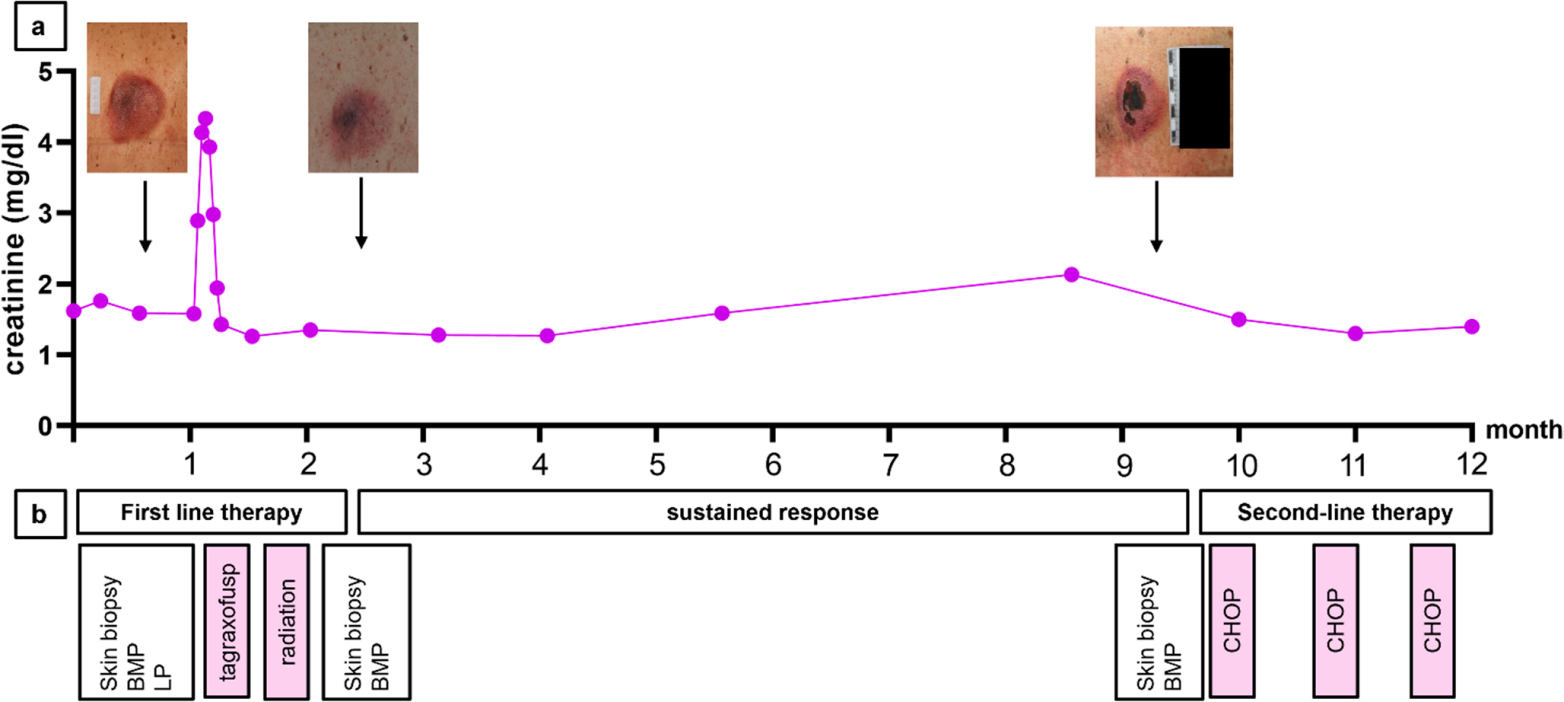
Time course of (**a**) creatinine levels and skin lesion in relation to (**b**) BPDCN treatment and remission status. After the diagnosis of BPDCN, the patient received dose-reduced tagraxofusp. Following three infusions, he developed acute-on-chronic renal failure, and tagraxofusp was discontinued. Under supportive management with fluid balance and prednisolone, renal function rapidly returned to baseline without the need for dialysis. After consolidative radiation of the skin lesion, re-biopsy confirmed complete remission of the skin lesions, while bone marrow residual infiltration was < 5%. The patient maintained a sustained response and excellent clinical condition for eight months, until hematologic and cutaneous relapse occurred. Following three cycles of full-dose CHOP, a very good partial remission was again achieved. **a** was created using GraphPad Prism v8 (GraphPad Software, La Jolla, CA)

The patient was transitioned into a watch-and-wait approach in good clinical condition (ECOG 1) and scheduled for regular follow-up visits. Eight months later, worsening blood values were noticed, including rapidly progressive thrombocytopenia, anemia, leukocytosis and rising creatinine levels. Manual differential blood counts indicated peripheral circulation of blasts. A bone marrow biopsy confirmed relapsed BPDCN with > 90% bone marrow infiltration. Additionally, local recurrence with central necrosis was detected at the original skin lesion site on the lower back (Fig. 3a). We carefully considered re-exposure to tagraxofusp but decided against it due to the patient’s prior renal injury, his preference to minimize further hospitalizations, and markedly higher tumor burden at relapse, which could increase the risk of capillary leak syndrome. Instead, conventional outpatient chemotherapy was selected to optimize both safety and therapeutic efficacy. After three cycles of CHOP, the patient achieved complete remission of the bone marrow and a very good partial remission of the skin. In the follow-up bone marrow panel, the KRAS mutation was no longer detected, while the CBL, TET2 and ZRSR2 mutations were still present, which could be attributed to the residual MDS. Creatinine levels returned to baseline, suggesting that the transient increase had been related to tumor lysis of blasts. At the most recent follow-up, 13 months after initial diagnosis, the patient remained in good overall condition, with stable renal function, preserved independence in daily activities, and no new clinical concerns. After completion of the fourth chemotherapy cycle, the patient was transitioned to surveillance.

## Discussion

BPDCN is a rare and aggressive hematologic malignancy that presents significant therapeutic challenges [12]. Tagraxofusp, a CD123-directed cytotoxin, has emerged as a promising targeted therapy for BPDCN, demonstrating encouraging efficacy in real-world cases [6, 10]. However, data on the tolerability and efficiency of tagraxofusp in elderly patients with significant co-morbidities such as cardiovascular disease or chronic kidney failure remains limited, highlighting the need for cautious evaluation and personalized treatment strategies [9].

One of the primary concerns with the use of tagraxofusp is its potential to cause CLS, particularly relevant for elderly patients, who may have a diminished ability to tolerate the fluid shifts associated with CLS [8]. The overall incidence of CLS in clinical trials was 55%, with 9% of patients experiencing Grade 3 events and 1% experiencing Grade 4 events. The median time to onset was 4 days, and all but 5 patients experienced the event in Cycle 1 [6]. CLS can exacerbate underlying renal dysfunction, leading to acute-on-chronic renal failure, a significant risk for patients with preexisting kidney impairment [13]. While clinical trials have demonstrated the efficacy of tagraxofusp, patients with CKD were generally excluded from these studies [6]. As a result, there is no published consensus on the safety of tagraxofusp in this specific patient population. The lack of data regarding pharmacokinetics and optimal dosing in patients with renal dysfunction presents a gap in our understanding of how to best manage this BPDCN population. In this case report, we highlight a patient with BPDCN who also had preexisting CKD and multiple comorbidities. The patient was treated with a reduced, half-cycle regimen of tagraxofusp, as the maximum tolerated dose. Despite the presence of a TET2 mutation, considered as an adverse prognostic marker in BPDCN [14], less than one cycle low-dose tagraxofusp elicited a notably prolonged response with intermittent dermatologic complete response. Although the patient subsequently received local radiation to the skin lesion, clinical macroscopic assessment prior to radiation already indicated substantial improvement consistent with clinical remission, primarily attributable to minimal-dose tagraxofusp. However, because no biopsy was performed between the completion of tagraxofusp and radiation, the extent to which histologic complete remission can be attributed solely to tagraxofusp remains uncertain, and radiation may have contributed to the final histologic outcome.

Mechanistically the molecular profile of the patient harboring mutations in CBL, KRAS, TET2, and ZRSR2 might explain the durable response to tagraxofusp. While TET2 and RAS pathway mutations are commonly observed in BPDCN, mutations in CBL and ZRSR2 occur less frequently [15]. Tagraxofusp requires high CD123 expression and receptor-mediated internalization to deliver its diphtheria toxin payload effectively [7]. However, CBL proteins tag activated receptor-associated kinases and adaptor proteins for ubiquitination, leading to their degradation [16]. Thus, CBL loss-of-function may stabilize CD123 on the cell surface, enhancing the uptake and efficacy of tagraxofusp therapy. TET2 loss fosters an aberrant epigenetic state that reinforces a plasmacytoid dendritic cell–like program, where high CD123 expression is a lineage hallmark [14]. Similarly, ZRSR2 mutations impair plasmacytoid dendritic cell activation and differentiation, preserving an immature, CD123-rich phenotype [17]. KRAS mutations are recognized driver events in BPDCN that promote proliferation through MAPK signaling [18]. Notably, in this patient, the KRAS mutation was no longer detectable at remission, while CBL, TET2, and ZRSR2 mutations persisted. This might represent a case of BPDCN arising from clonal hematopoiesis, a concept that has previously been demonstrated for BPDCN [19] and supports the interpretation that KRAS acted as a disease-driving alteration in this case. Taken together, the molecular profile may have actively supported a biological state characterized by sustained CD123 expression and heightened sensitivity to tagraxofusp, providing a plausible explanation for the sustained response observed in this patient.

This case is unique in representing the first reported instance of successful tagraxofusp therapy in a patient with advanced chronic kidney disease (CKD), providing valuable insights into the potential use of this treatment in populations highly susceptible to adverse events. Despite achieving complete remission, the development of acute-on-chronic renal failure following the third infusion underscores the importance of vigilant monitoring and individualized treatment strategies in such high-risk patients. At relapse, the patient received four cycles of CHOP chemotherapy, which proved effective, resulting in persistent remission several months after treatment cessation, highlighting that BPDCN remains sensitive to conventional chemotherapy even after initial immunotherapy. In conclusion, while tagraxofusp represents a promising treatment for BPDCN, its use in patients with CKD requires careful consideration. Further clinical studies are needed to evaluate safety, efficacy, and optimal dosing in this subgroup. Until more data are available, tagraxofusp should be used with caution in patients with renal impairment, with treatment regimens carefully adjusted and closely monitored to minimize the risk of severe adverse events.

## Data Availability

No datasets were generated or analysed during the current study.
